# EHMTI-0316. Exam findings predict outcome of C1 block for migraine treatment

**DOI:** 10.1186/1129-2377-15-S1-D31

**Published:** 2014-09-18

**Authors:** M Johnston, SE Jordan, AC Charles

**Affiliations:** 1 Neurology, University of California Los Angeles, Los Angeles, USA

## Introduction

Previous studies have shown the C1 spinal nerve has sensory neurons. Direct stimulation of the C1 spinal nerve provokes peri-orbital pain in migraine patients. No data exist which can predict a positive outcome for C1 nerve root block. Tenderness over the greater occipital nerve has been shown to predict outcome of GON block. We propose that tenderness over the GON with periorbital referral on exam predicts periorbital referral on direct C1 stimulation and predicts a positive outcome of block.

## Aims

Predict the outcome of C1 spinal nerve block based on exam findings.

## Methods

Review of 23 patients, 21 of whom had chronic migraine and 2 of whom had chronic cluster headache. All 23 have undergone C1 spinal nerve block.

## Results

Of the 23 patients, 17 (74%) had GON tenderness on exam with periorbital referred pain and 6 (26%) had only occipital tenderness. Both cluster patients did not have periorbital nor orbital pain on palpation of the GON. All 17 with periorbital referred pain on GON palpation had reproducible periorbital pain intraoperatively on direct stimulation of the C1 spinal nerve with fluoroscopic guidance. Of those, 15/17 (88%) had a positive block. Of the 6/23 (26%) with a negative block, only two had periorbital pain reproduced on exam by GON palpation. Both cluster headaches had negative blocks. Neither had intraoperative periorbital or orbital pain on C1 stimulation.

## Conclusion

Tenderness over the GON with periorbital pain referral during exam predicts positive outcome of C1 spinal nerve block in patients with migraine.

No conflict of interest.

